# Variation in pickleweed root-associated microbial communities at different locations of a saline solid waste management unit contaminated with petroleum hydrocarbons

**DOI:** 10.1371/journal.pone.0222901

**Published:** 2019-10-03

**Authors:** Abdur Rahim Khan, L. G. Reichmann, J. C. Ibal, J. H. Shin, Y. Liu, H. Collins, B. LePage, N. Terry

**Affiliations:** 1 Department of Plant and Microbial Biology, University of California, Berkeley, CA, United States of America; 2 School of Applied Biosciences, College of Agriculture and Life Sciences, Kyungpook National University, Daegu, Republic of Korea; 3 USDA-ARS Grassland Soil and Water Research Laboratory, Temple, TX, United States of America; 4 Pacific Gas and Electric Company, San Ramon, CA, United States of America; 5 The Academy of Natural Science, Philadelphia, PA, United States of America; Universidade de Coimbra, PORTUGAL

## Abstract

The main purpose of this study was to explore the potential influences of pickleweed vegetation on the abundance, diversity and metabolic activities of microbial communities in four distinct areas of a petroleum-contaminated solid waste management unit (SWMU) located in Contra Costa County, northern California. The four areas sampled include two central areas, one of which is central vegetated (CV) and one unvegetated (UV), and two peripheral vegetated areas, one of which is located to the west side of the SWMU (V-West) and one located to the east side (V-East). Measurements were made of total petroleum hydrocarbons (TPH), polyaromatic hydrocarbons (PAH), soil physicochemical properties, and various aspects of microbial communities including metabolic activities, microbial abundances (PLFAs), diversity and composition based on amplicon sequencing. The peripheral V-East and V-West sites had 10-times lower electrical conductivity (EC) than that of the CV and UV sites. The high salinity levels of the CV and UV sites were associated with significant reductions in bacterial and fungal abundances (PLFA) when compared to V-East but not when compared to V-West. TPH levels of CV and UV were not significantly different from those of V-West but were substantially lower than V-East TPH (19,311 mg/kg of dry soil), the high value of which may have been associated with a pipeline that ran through the area. Microbial activities (in terms of soil respiration and the activities of three soil enzymes, i.e., urease, lipase, and phosphatase) were greatest in the vegetated sites compared to the UV site. The prokaryotic community was not diverse as revealed by the Shannon index with no significant variation among the four groups of samples. However, the fungal community of the peripheral sites, V-East and V-West had significantly higher OTU richness and Shannon index. Structure of prokaryotic communities inhabiting the rhizosphere of pickleweed plants at the three sites differed significantly and were also different from those found in the UV region of the central site according to pairwise, global PERMANOVA and ANOSIM analyses. The differences in OTU-based rhizosphere-associated bacterial and fungal communities’ composition were explained mainly by the changes in soil EC and pH. The results suggest that saline TPH-contaminated areas that are vegetated with pickleweed are likely to have increased abundances, diversity and metabolic activities in the rhizosphere compared to unvegetated areas, even in the presence of high salinity.

## Introduction

Owing to industrial runoffs, effluent releases and accidental spills, many places around the world are contaminated by total petroleum hydrocarbons (TPHs) [1]. The magnitude of worldwide contamination by petroleum-derived products is such that TPHs are considered to be the most widespread class of organic contaminants [2]. TPHs accumulate in soil and can remain over long periods [3]. Because of their toxicity, mutagenicity, and carcinogenicity, TPHs may have significant effects on the microbiota and other ecosystem components and pose serious health risks to humans and animals. The development of effective strategies for the decontamination of TPH-polluted soils is of vital importance. Thermal, chemical, and physicochemical technologies have been developed and applied to remediate soils contaminated by TPHs [4]. However, these approaches are expensive and require the use of heavy machinery, which may result in significant disturbances of the site [5]. A less expensive and more environmentally acceptable approach to remediation is bioremediation–a process that employs the use of both plants and microbes to clean up TPH-contaminated soil [6] [7]. The joint action of the plant and the microbiota associated with its roots has been shown to be effective in the degradation of organic pollutants in the rhizosphere [8]. Plants enhance the abundance, diversity and catalytic activities of soil rhizospheric microorganisms by the plant roots primarily through the exudation of a variety of organic compounds [9].

Petroleum hydrocarbon contamination often occurs in areas of high salinity [10]. In such saline environments, the implementation of effective rhizoremediation strategies is more challenging since high levels of soil salinity strongly inhibit the germination and growth of plants and also affects soil microbial activities [11]. The use of halophytic plant species may help overcome some of the limitations associated with the bioremediation of saline soils contaminated with petroleum hydrocarbons (PHs). In this context, phytoremediation studies using halophytic plants such as triangular club-rush (*Scirpus triqueter*), sea purslane (*Halimione portulacoides)*, (*Suaeda salsa)*, and sea rush (*Juncus maritimus)* have provided promising results [11, 12]. Different halophytic plant species differ greatly with respect to their root morphology and the nature of their root exudates; this in turn influences the rhizospheric microbial communities and the efficiency with which PHs can be degraded in the rhizosphere [11]. However, it is unclear as to the extent to which rhizospheric microbial communities of one specific halophyte plant species differs spatially within the same contaminated area, and which environmental factors could potentially explain these differences. The spatial variability of rhizospheric microbial communities may also be a consequence of changing root ages and root exudate composition [13]. It is therefore important to investigate the potential spatial differences of rhizospheric microbial communities that influence the bioremediation rate of the contaminants at a specific site.

Pickleweed (*Salicornia virginica*) is a perennial succulent halophytic herb and a major component of wetland communities in San Francisco Bay salt marshes. Pickleweed has previously been used for the bioremediation of saline PH-contaminated soils [14]. In the present study, we aimed to compare the rhizospheric microbial communities of pickleweed plants with those inhabiting the bulk soils (unvegetated) located in Contra Costa County, northern California–a site which is saline and contaminated with petroleum hydrocarbons [15]. We investigated microbial communities associated with the rhizosphere of pickleweed-plants (vegetated) and bulk soil (un-vegetated) in terms of microbial abundance, activities, taxonomic diversity and composition to answer the following research questions: 1) Do rhizosphere-associated microbial communities of pickleweed plants growing in three different zones of a petroleum hydrocarbon-contaminated soil site differ? 2) Are pickleweed rhizosphere-associated microbial communities different from those inhabiting the bulk soil? 3) What are the main soil physicochemical factors that influence the microbial communities at this site?

## Materials and methods

### Site description and sampling

**Fig 1:**
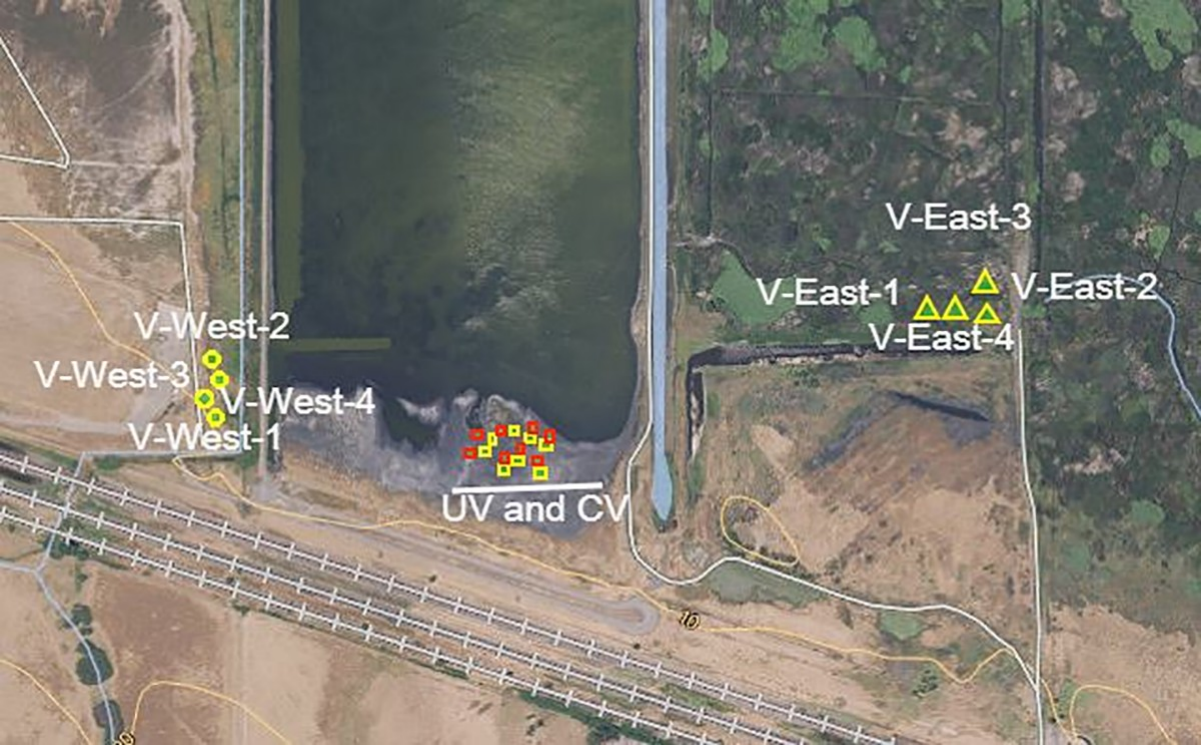
Schematic representation of the sampling sites within solid waste management unit (SWMU) located in Contra Costa County, northern California. A total of twenty-four composite samples were taken at four different areas: 1) Total of eight composite rhizosphere-associated soil samples from pickleweed plants growing on the east (4-samples) and west side (4-samples) of the central SWMU area and referred as peripheral vegetated, V-East (yellow triangles), and V-West (yellow stars) sites, respectively. 2) From the central SWMU sampling site; eight rhizosphere-associated soil samples from pickleweed plants growing within SWMU site (CV, yellow rectangular); and eight bulk soil (un-vegetated) samples within SWMU site away from pickleweed plants rhizosphere (UV, red rectangular).

The experimental sampling area is a 29-ha solid waste management unit (SWMU) located in Contra Costa County, northern California. The Pacific Gas and Electric (PG&E) Company owns this site and granted permission for sampling. The field studies did not involve any endangered or protected species. The site is contaminated with petroleum hydrocarbons, polyaromatic hydrocarbons (PAHs), and some heavy metals from past industrial activities. Most contaminants are located in the upper layer of the site (15 to 60 cm depth), which is sporadically colonized by native plants [15]. For the current study, we selected two central areas; central vegetated (CV) and unvegetated (UV) sites in the SWMU and two peripheral vegetated areas (V-East and V-West sites) outside of the SWMU for collecting samples (Fig 1). A total of 24-composite soil/waste samples were collected across the four sites. Each of the root-associated composite samples (CV, V-East and V-East samples) consisted of 3-subsamples of soil. Soils, along with plant roots, were collected within 3 cm of a stem with a spatula to 10 cm deep. Each subsample was taken from the soil surrounding what we thought was the same modular pickleweed plant, so samples were up to 2 or 3 meters apart from one another [16]. Each UV-composite waste sample consisted of 3-subsamples collected from unvegetated soils, at least 2 to 3 meters apart from a plant. Composite samples were within a radius of 1 to 1.5 meters. The distribution of the 24 composite samples was as follows: 1) four composite soil samples were collected from the V-East site; 2) four composite soil samples collected from the V-West site; 3) eight composite waste samples were taken from the CV site and eight composite waste samples were taken from UV site in the SWMU. Samples were transferred in Zip Freezer bags on dry ice to the laboratory, where the soil was removed from the root fragments by shaking vigorously and root fragments were discarded. Soil samples were stored at 4°C under aerobic conditions for physicochemical, TPH content, and microbial activity measurements or stored at -80°C prior to phospholipid fatty acids (PLFA) analysis and Ion Torrent amplicon sequencing.

### Physicochemical characterization of soil and waste sediments

*In situ* measurements of the volumetric water content of the soil (m^3^/m^3^), electrical conductivity (bulk EC, mS/cm), and temperature (°C) were measured using a handheld reader connected to a soil sensor (Decagon´s GS3 Greenhouse Sensor and ProCheck sensor reader). Soil pH and water potential were measured in the laboratory using an EC500 meter (ExStick II, Extech Instruments) and a dewpoint potential meter (WP4, Decagon Devices Inc). Soil inorganic N content (NH_4_^+^ and NO_3_^-^) was measured using standard colorimetric methods [17, 18]. About 3 g of soil/waste was mixed with 20 ml of 2M KCl solution. After shaking for 1h, the filtered extracts were used for determination of inorganic N. Another soil subsample was placed in a drying oven at 105°C for 48h for the estimation of gravimetric soil water content and for correcting the soil N concentration [19].

### Total petroleum hydrocarbon contents

To determine the TPH composition and concentration in the different samples, 5–10 g of soil/waste from each sample was oven-dried at 60°C, ground and extracted with 150 ml of dichloromethane, hexane, and chloroform (1:1:1, Soxhlet-extraction) for 18h at 54°C. Extracts were dried at room temperature by evaporating the solvents under a gentle air stream in the fume hood. The amount of residual TPH recovered was determined gravimetrically [20]. Just before the gas chromatography-mass spectrometry (GC-MS) analysis, the TPH samples were re-suspended in 1 ml chloroform containing 50 μg of O-terphenyl as internal standard and stored in sealed glass vials at -20°C. The concentrations of TPHs and PAHs were determined using GC-MS (SHIMADZU GCMS-QP2010, Japan) under the following conditions: helium (99.99% purity) as the carrier gas at a constant flow rate of 11.5 ml/min, column flow rate of 1.00 mL/min, purge flow rate of 0.5 mL/min, linear velocity of 36.1 cm/s and a pressure of 51.2 kPa. Samples were injected onto a 30 m ZB-5HT INFERNO column (Zebron, United States) with a 0.25 mm i.d. and 0.25 μm film thickness. One microliter aliquots of each chloroform extract was injected at 280°C and the injection mode was split at a ratio of 10:1. The oven temperature of GC was held at 45°C for 7min, and then increased by 5°C/min from 45 to 180°C, followed by an increase to 380°C (35°C/min), held for 3min, and then decreased to 50°C (60°C/min). In the selected ion monitoring (SIM) mode, about 150 target compounds of different classes including alkanes, PAHs, BTEX, and cycloalkane were detected. The relative abundance of different compounds was calculated as the ratio of the peak area of each hydrocarbon to the peak area of the internal standard.

### Soil microbial activities

Microbial community was characterized by measuring soil basal respiration and three potential enzyme activities (lipase, urease, and phosphatase). Soil basal respiration was measured using the alkali absorption method [21]. Briefly, 10 g of soil/waste from each sample was incubated in a 1L glass jar that contained NaOH solution in a vial to absorb the CO_2_ produced during incubation. After 24h of incubation at a temperature of 22°C, 2 ml of BaCl_2_ was added to the NaOH, and the excess hydroxide was titrated with 0.1 M HCl in the presence of phenolphthalein as an indicator. Lipase activity was determined calorimetrically by using p-nitrophenyl (pNP) butyrate as a substrate according to the method described by [22]. Soil/waste samples were incubated at 30°C and pH 7.0 for 10min and the released pNP was quantified spectrophotometrically at 400 nm. Urease activity (E.C. 3.5.1.5) was analyzed using the procedure developed by Kandeler and Gerber [23]. Briefly, 2.5 g of fresh soil/waste was incubated with 1.25 mL of 0.08M aqueous urea solution for 4 h at 30°C. The NH_4_^+^- produced was extracted with 1 M KCl and 0.01 M HCl and quantified by means of a modified indophenol reaction. Alkaline phosphatase activity (EC 3.1.3.1) was carried out following the methodology described by Eivazi and Tabatabai [24] using 0.115 M p-nitrophenyl phosphate as a substrate and a 0.5 M sodium acetate-acetic acid buffer with a pH of 8.5. After 1 h of incubation at 20°C, the pNP released during incubation was extracted and colorimetrically determined at 400 nm.

### Microbial abundance

Phospholipid fatty acids were extracted and analyzed from each sample following the method described by Buyer and Sasser [25]. Briefly, microbial lipids were extracted from 2 g of lyophilized soil sample using 3 mL of Bligh–Dyer buffer (10:4:5, methanol:chloroform:phosphate). Simultaneously, 1 mg of 19:0 phosphatidylcholine (Avanti Polar Lipids, Albaster, AL) was dissolved in 25 mL of Bligh-Dyer extractant and added to each sample (100 μL) as an internal standard. Samples were vortexed for 5 seconds and sonicated in a water bath for 10min for three times. The samples were then centrifuged at 20°C and 3500×g for 15 min. Following centrifugation, the organic molecules were extracted from the supernatant via liquid-liquid extraction with 1 mL each of chloroform and deionized water. Lipids were separated from the resulting organic layer via solid-phase extraction with a 96-well SPE plate (HyperSep 50mg/1mL, Thermo), transesterified and quantified by GC-MS.

### Microbial community analysis

Total genomic DNA was extracted in duplicate (24 samples × 2 DNA extractions = 48 DNA extracts) from 250 mg soil/waste aliquots using the DNeasy PowerSoil Kit (Qiagen, Valencia, CA) according to the manufacturer's instructions. Then, duplicate DNA extracts from each sample were pooled and the quality of the DNA checked using electrophoresis and spectrophotometer. The extracted DNA was used as template for amplification of the V4–V5 regions of the prokaryotic 16S rRNA gene and the fungal internal transcribed spacer (ITS) 2 region by polymerase chain reaction (PCR). For the prokaryotic PCR, we used the universal prokaryotic primers 515F (5′ GTGCCAGCMGCCGCGGTAA -3′) and 907R (5′ CCGYCAATTCMTTTRAGTTT -3′). The fungal ITS2 region was amplified by PCR using ITS86F (5’–GTGAATCATCGAATCTTTGAA–3’) and ITS4R (5’–TCCTCCGCTTATTGATATGC–3’) primers. The PCR mixtures and cycling conditions were as reported previously [26, 27].

The amplicons for both prokaryotic and fungal communities were sequenced using the Ion Torrent PGM sequencing platform following the protocols provided in the manual. The raw FASTQ files were first demultiplexed and quality-filtered using Trimmomatic [28] and subsequently processed using QIIME (version 1.9.1) [29]. After chimeras were removed, prokaryotic and fungal sequences were independently clustered into OTUs at a 97% sequence identity and taxonomically affiliated using Versions Greengenes database (V13.8) for the 16S rRNA genes and UNITE (Version 7.2) reference databases, respectively, with a 50% confidence threshold. One OTU table was generated for each microbial community and the number of sequences per sample was normalized to 4,242 and 4,443 for prokaryotes and fungi, respectively. These two normalized OTU tables were used for downstream analyses. Richness and Shannon index were calculated using Mothur v.1.39.5 [30].

### Data analysis

We performed type II ANOVA (analysis of variance) to test for differences in (i) soil physicochemical properties, (ii) TPH content, (iii) potential enzyme activity, (iv) PLFA-based abundance of microbial communities, (v) diversity measurements of soil microbial communities, and (vi) relative abundance of the different taxonomic groups among the samples investigated. When ANOVA yielded significant results, Tukey’s HSD (honest significance difference) post-hoc test was used for multiple comparisons of means at a 95% confidence interval. Normality and heteroscedasticity of data were tested by the Shapiro-Wilk and Levene tests, respectively. In case that one of these conditions was not met, the values were transformed using natural logs.

Non-Metric Multi-Dimensional Scaling (NMDS) was chosen as the ordination method to visualize patterns in OTU-based prokaryotic and fungal community structures. The significance of the variations in the composition of microbial communities was tested by means of PERMANOVA (permutational analysis of variance) and ANOSIM (analysis of similarity) with Bray-Curtis dissimilarities and 9,999 permutations. All the analyses were done using the package “Vegan” in R. SIMPER (similarity percentage) analysis was applied to identify the prokaryotic and fungal OTUs primarily responsible for the observed dissimilarities among the rhizospheric samples from the three sites investigated using Bray-Curtis dissimilarities with the software PAST ver. 3.07 [31]. A Mantel test, which compares the Bray-Curtis dissimilarities matrices with 9,999 permutations, was used to investigate the physicochemical factors of the samples and in order to explain the differences in the composition of prokaryotic and fungal communities in the samples.

## Results

### Soil physicochemical characteristics and total petroleum hydrocarbon contents

All the physicochemical characteristics (except N-NH_4_^+^ and N-NO_3_^-^ contents) of the pickleweed rhizosphere-associated soil samples of the three studied zones were significantly different (Table 1). The highest values for electrical conductivity (EC) were observed in the central CV and UV sites compared to the V-East and V-West sites located outside of the SWMU. The V-West site had significantly lower soil water potentials (WP) compared to other sampling sites. The concentration of TPHs were approximately 3-fold higher in the vegetated V-East site than the V-West, CV, and UV sites (**Table 1**). The distribution of the TPHs classes showed a dominance of n-alkanes at all sites, with smaller contributions of PAHs, alkanes, and cycloalkanes to the total TPHs in soil (**S1 Fig)**. When looking at the hydrocarbon size, we found that more than 60% of the hydrocarbons remaining in the soil belonged to high molecular weight carbon compounds (i.e., C number of 25 or more) (**S2 Fig**).

**Table 1:** Physicochemical differences among the sample sites.

Variable	V-East (N = 4)	V-West (N = 4)	CV (N = 8)	UV (N = 8)	ANOVA (p>F)
Electrical Conductivity (dS m^-1^)	1.29 ± 0.91 (b)	0.39 ± 0.50 (b)	10.61 ± 5.76 (a)	12.30 ± 2.81 (a)	***
% Gravimetric Water	47.26 ± 22.81 (a)	4.43 ± 2.12 (b)	59.11 ± 25.14 (a)	39.26 ± 6.20 (a)	***
Water potential (MPa)	-0.26 ± 0.14 (a)	-52.02 ± 9.44 (b)	-7.63 ± 3.01 (c)	-3.90 ± 1.98 (ac)	***
pH	7.41 ± 0.35 (b)	7.78 ± 0.10 (ab)	8.08 ± 0.11 (a)	7.41 ± 0.45 (b)	**
N-NH_4_^+^ (μg kg-1 soil)	7.94 ± 5.28	3.50 ± 0.71	7.13 ± 2.82	4.05 ± 2.91	NS
N-NO_3_^-^ (μg kg-1 soil)	3.56 ± 3.34	0.52 ± 0.56	0.94 ± 1.57	1.78 ± 2.23	NS
TPHs (mg kg^-1^ soil)	19,311 ± 3,879 (a)	5,975± 3,3133 (b)	5,400 ± 1,853 (b)	9,422 ± 6,973 (b)	**
Respiration (μg CO_2_ g^-1^ soil h^-1^)	19.92 ± 5.83 (ac)	21.63 ± 4.59 (a)	14.62 ± 2.23 (cb)	10.44 ± 3.61 (b)	***
Lipase (μg p-nitrophenol g^-1^ soil h^-1^)	1,195 ± 350 (ac)	1,298 ± 275 (c)	744 ± 303 (ad)	626 ± 216 (bd)	**
Urease (μmol NH4/g^-1^ soil h^-1^)	1,306 ± 187 (a)	951 ± 328 (ac)	1,062 ± 253 (a)	608 ± 184 (c)	***
Phosphatase (μmol p-nitrophenol g^-1^ soil h^-1^)	1.74 ± 1.01 (a)	0.58 ± 0.13 (bc)	1.43 ± 0.49 (ac)	0.45 ± 0.24 (b)	***
Total PLFAs (nmol g^-1^ of dry soil)	1,336 ± 625 (a)	235 ± 66 (b)	583 ± 505 (b)	188 ± 102 (b)	***
Bacteria PLFA	985 ± 460 (a)	172 ± 53 (b)	432 ± 349 (b)	164 ± 89 (b)	***
Fungi PLFA	310 ± 143 (a)	55 ± 16 (b)	130 ± 140 (b)	21 ± 12 (b)	**
Bacteria: Fungi PLFA ratio	3.18 ± 0.25 (a)	3.10 ± 0.71 (a)	3.66 ± 0.76 (a)	7.90 ± 2.43 (b)	***

[Note: Different letters correspond to differences between sites for the particular variable (Tukey multiple comparisons of means; 95% family-wise confidence level). Values correspond to mean ±1 standard deviation. Significance codes

‘***’ <0.001

'**' <0.0].

### Soil microbial activities

The activity of microbial communities was determined by measuring the basal respiration and three potential enzyme activities (lipase, urease, and phosphatase). These parameters were all substantially higher in vegetated locations compared to un-vegetated locations (**Table** 1**)**. The highest values for basal respiration and lipase activity were found in V-West samples, while the V-East samples presented the highest values for urease and phosphatase activity.

### Microbial abundance

Phospholipid fatty acid analyses (PLFAs) showed the total microbial, bacterial, and fungal abundances were significantly greater at the V-East site. However, the PLFA abundances did not differ significantly among the pickleweed vegetated sites. The UV site had a significantly higher proportion of bacterial to fungal PLFAs than the other sites, while this ratio was similar across the CV, V- East, and V-West site (**Table** 1).

### Richness and diversity of soil microbial communities

**Fig 2:**
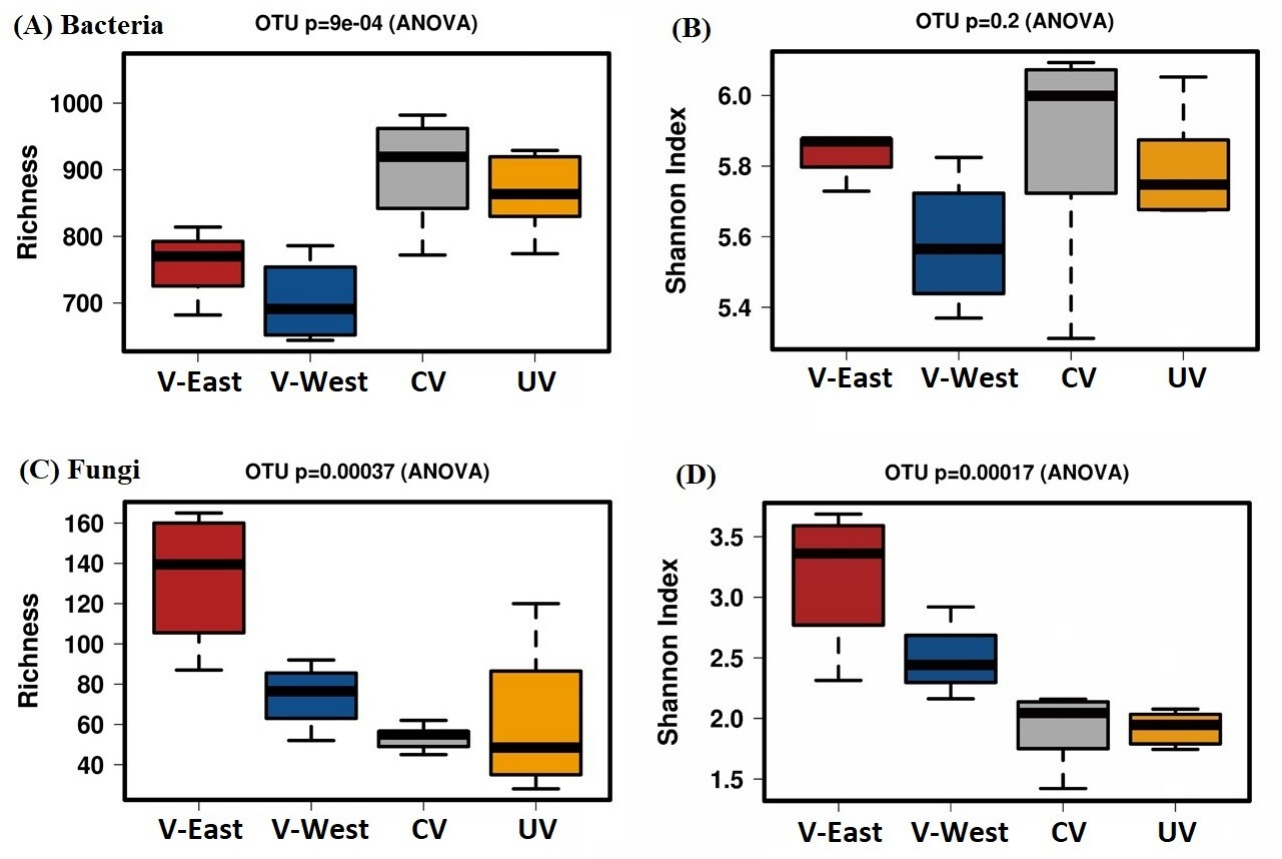
Richness and Shannon index represent alpha diversity of bacterial and fungal communities of the four sites. The number of OTUs were used as a measure of the richness and Shannon index for both prokaryotic (**A, B**) and fungal (**C, D**) communities in the peripheral vegetated, V-East and V-West sites and the central vegetated (CV) and the un-vegetated (UV) sites. Lower, mid, and upper horizontal lines represent the first quartile, median and the third quartile, respectively.

Across the 24 samples analyzed, we obtained a total of 526,139 high-quality 16S rRNA reads distributed among 2,534 different OTUs. In fungi, sequencing analysis revealed that there were 253,805 high-quality reads distributed among 310 OTUs. Venn diagrams revealed 133 prokaryotic OTUs (representing 5% of the OTUs) were shared among all the vegetated sites (V-East, V-West and CV), while 533 OTUs (21%) were shared by UV and CV sites (**S3A Fig**). Among the fungi, 9 OTUs (3%) were shared by all vegetated sites and 51 OTUs (21%) were common for the UV and CV sites (**S3B Fig**). The soil samples at site V-East presented the higher number of unique OTUs (16%). Although prokaryotic richness was significantly higher in the CV and UV samples in comparison with the samples at the V- East and V-West sites, the Shannon index did not differ significantly among the different samples (**Fig 2A and 2B**). For the fungi, the V- East and V-West sites presented significantly greater values for OTU richness and Shannon index (**Fig 2C and 2D)** compared with the CV and UV sites.

### Changes in soil microbial community structure

**Fig 3:**
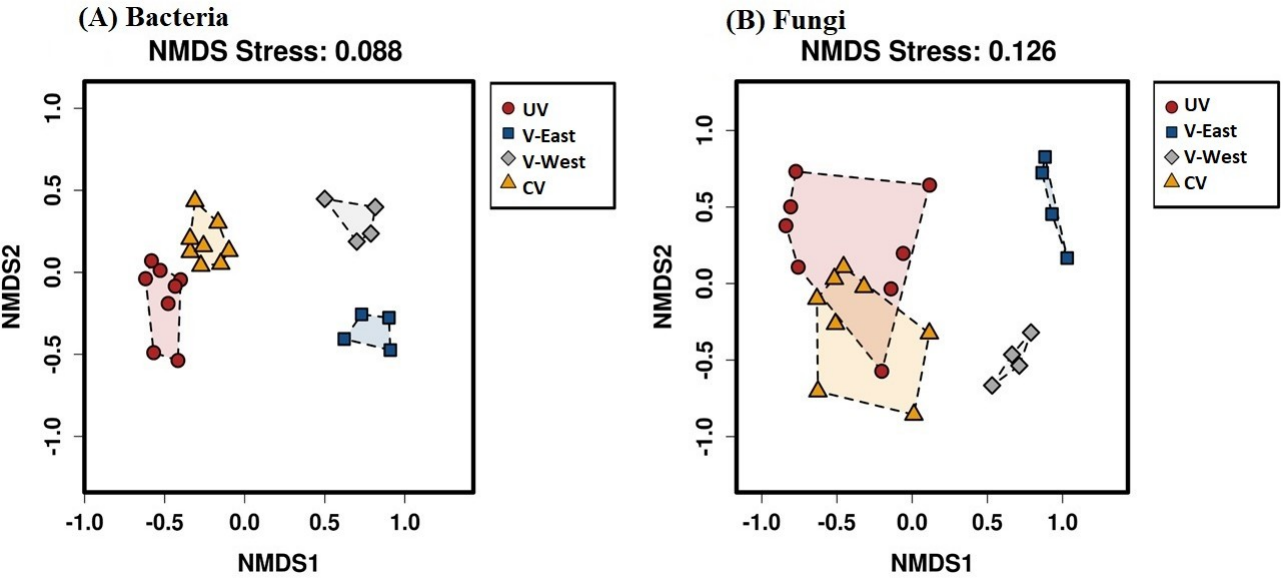
Non-metric multidimensional scaling (NMDS) plot displaying the community composition assignments of: (A) prokaryotic, and (B) fungi cross four sites. NMDS ordination of 24 samples across four sites was carried out based on Bray-Curtis dissimilarity distances calculated from pairwise taxonomic profile comparisons between all samples. A shorter linear distance between two samples denote greater similarity between the corresponding samples. Samples from four locations are presented by different colors (see legend).

The structure of the prokaryotic communities from the CV, V-East, and V-West sites differed significantly from UV samples according to pairwise (**S1 and S2 Tables**), global PERMANOVA (F = 6.43; p = 0.0001) and ANOSIM (R = 0.831; p = 0.0001) results. This was shown graphically using NMDS (**Fig 3A**). The NMDS axis 1 separated the V-East and V-West samples from the CV and UV samples. The NMDS axis 2 further separated the CV and UV sample cluster and the V-East and V-West cluster. SIMPER pairwise comparisons (considering all the groups of samples) demonstrated that bacterial community composition dissimilarity was the lowest (63.1%) between the UV and CV samples.

The fungal NMDS clustered the samples into three groups; one comprising the CV and UV samples and the V-East and V-West samples. Global PERMANOVA (F = 7.558; p = 0.0001) and ANOSIM (R = 0.6531; p = 0.0001) results demonstrated the significance of this ordination. Pairwise PERMANOVA and ANOSIM (**S3 and S4 Tables**) showed the fungal community compositions at the CV, V-East, and V-West sites were significantly different. Likewise, the structure of the V-East and V-West fungal communities were significantly different from those at the CV sites; however, the structure of the CV and UV fungal communities did not differ significantly (**S3 and S4 Tables**). The fungal community composition dissimilarity (55.4%) value was lowest between CV and UV samples according to SIMPER pairwise comparisons.

**Fig 4:**
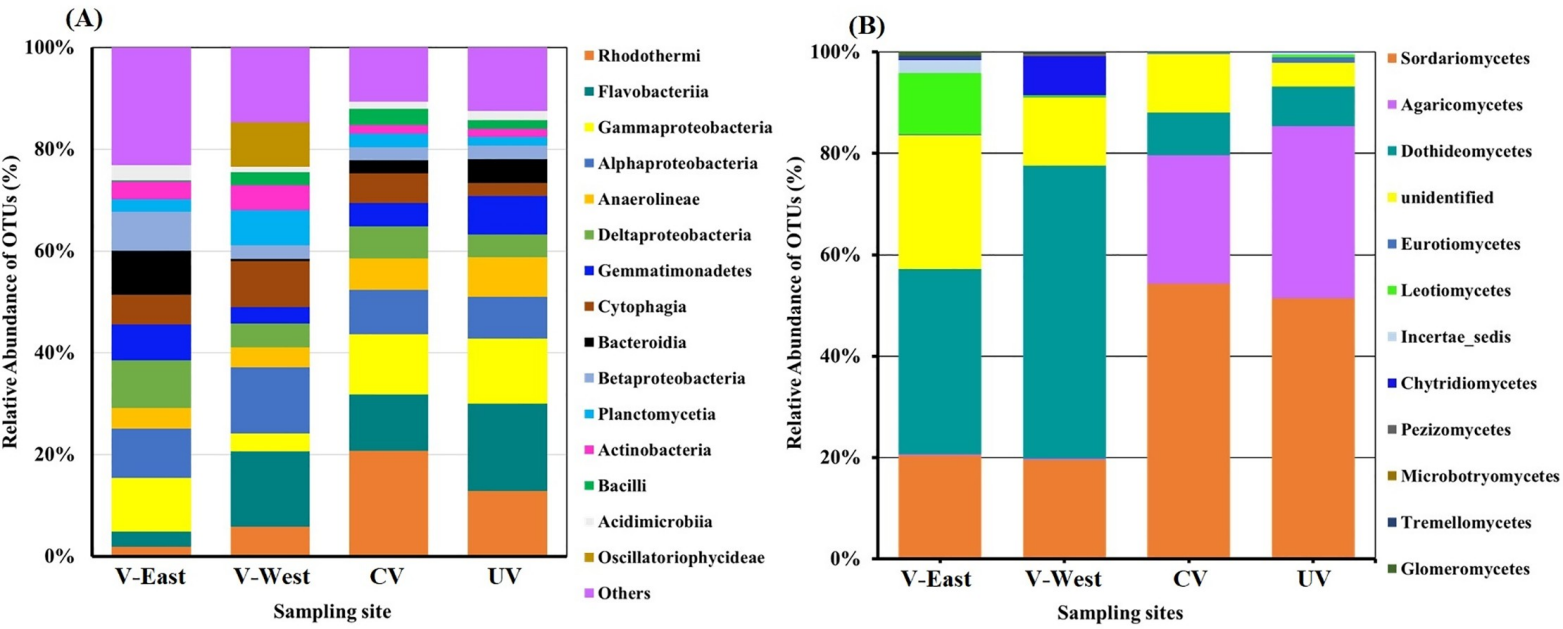
Mean relative abundance of the most abundant prokaryotic **(A)** and fungal classes (**B**) found in the peripheral vegetated, V-East and V-West sites and the central vegetated (CV) and un-vegetated (UV) zones from the central site.

Differences in the structure of prokaryotic and fungal communities among sites were also present at the taxonomic level (**Fig 4**). Total bacterial diversity was distributed across 28 different phyla, although the six most dominant phyla (*Bacteroidetes* (15.7–57.2%), *Proteobacteria* (17.5–43.1%), *Chloroflexi* (2.3–17.1%), *Gemmatimonadetes* (2.1–15.1%), *Actinobacteria* (1.7–9.0%), and *Cyanobacteria* (1.2–18.9%)) accounted for more than 73% of the total number of sequences (**Fig 4A**). *Deltaproteobacteria* and *Betaproteobacteria* (*Proteobacteria*) were significantly more abundant in the V-East site, whereas, *Planctomycetia* (*Plantomycetes*) and *Oscillatoriophycidea* (*Cyanobacteria*) were found to a significantly higher extent in the V-West site. The class *Rhodotermi* (*Bacteroidetes*) and *Bacilli* (*Fimicutes*) were dominant in the CV samples, while the class *Gemmatimonadetes* were found significantly more frequently in the UV samples **(S5 Table)**. In the SIMPER analysis, considering only the rhizosphere-associated samples (CV, V-East and V-West) showed that the top 50 prokaryotic OTUs only comprised 24.9% of the total dissimilarity. Some of these OTUs were successfully classified at genus level as *Phormidium* (*Cyanobacteria*), *Salinimicrobium*, *Balneola*, *Arenibacter* (*Bacteroidetes*), and *Haladaptatus* or *Halogranum* (*Halobacteria*, Archaea) (**S6 Table)**.

Overall, the composition of fungal communities across the 24 libraries analyzed was distributed among four phyla (*Ascomycota*, *Basidiomycota*, *Chytridiomycota*, and *Glomeromycota*) and one sub-phylum (*Mucoromycotina*). The *Ascomycota* (the proportion of the classified sequences in this phylum ranged from 18.2% to 90.3%) and *Basidiomycota* (0.4–79.9%) were predominant and a proportion of reads ranging from 0.2 to 35.1% across the 24 libraries were unclassified at phylum level. The *Sordariomycetes* (*Ascomycota*) were significantly more abundant (p < 0.05) in the V-East and V-West samples compared to the CV and UV samples. The opposite was noted for *Dothideomycetes* (**Fig 4B and S7 Table)**. A more detailed analysis of the different *Sordariomycetes* genera revealed fungi belonging to *Fusarium* were significantly predominant in the V-West samples, while *Scedosporium* were dominant in the CV and UV samples. *Acroconidiella* and *Alternaria* were prevalent in the CV and V-West samples, respectively. Interestingly, the number of unclassified sequences at the phylum level were significantly lower in the UV samples compared with the CV, V-East, and V-West samples. At the OTU level, SIMPER analysis showed that the top 20 fungal OTUs comprised 78.8% of the dissimilarity among the CV, V-East, and V-West samples. Some of these OTUs were classified at genus level as *Scedosporium*, *Alternaria*, *Acroconidiella*, *Phoma*, *Macrospora*, *Scytalidium*, *Myriococcum*, and *Rhizophydium* **(S8 Table).**

#### 3.6. Correlations between soil physiochemical properties and microbial community composition

According to the Mantel test, differences in the composition of rhizosphere-associated bacterial and fungal communities of the pickleweed plants at the three sites (CV, V-East, and V-West samples) could be attributed to variation in all of the soil physicochemical characteristics measured except water potential. The physicochemical factors contributing to the greatest extent that would explain these variations were EC and pH (**Table 2**).

**Table 2:** Mantel test results correlating OTU-based structure of soil prokaryotic and fungal communities with soil physicochemical properties in the vegetated soil at the peripheral sites, V-East and V-West and in the central vegetated (CV) and un-vegetated (UV) soils at the central SWMU site.

Variable	Bacterial communities		Fungal communities	
	R	p-value	R	p-value
Electrical Conductivity	**0.584**	0.0001	**0.442**	0.0007
% Gravimetric Water	**0.378**	0.0063	**0.283**	0.0164
Water potential (MPa)	-0.822	1	-0.714	1
pH	**0.536**	0.0001	**0.515**	0.0001
Temperature	0.224	0.0262	0.175	0.05
N-NH_4_^+^	**0.252**	0.0211	**0.211**	0.0366
N-NO_3_^-^	**0.344**	0.0094	**0.251**	0.0213
Total petroleum hydrocarbons	**0.397**	0.0015	**0.364**	0.0025

R2 values in bold indicate statistical significance (*p* ≤ 0.05).

## Discussion

Other researchers have shown that the plant rhizosphere-associated microbial communities influence (and are influenced by) a variety of environmental factors related to soil physicochemical characteristics, nutrient availability, and vegetation type [32–34]. Our investigation of the prokaryotic and fungal community diversity and composition associated with the rhizosphere of pickleweed plants growing within the SWMU contaminated with TPH, PAHs, and metals, as well as localities outside of the SWMU, revealed that plant colonization did not lead to significant changes in prokaryotic diversity measured using the Shannon index in the V-East, V-West, and CV sites. However, the structure of the bacterial communities at the V-East, V-West, were significantly different from CV sites as visualized by NMDS plots, which separated the V-East and V-West sites from that of the central CV site. The fungal communities at the V-East, V-West sites were more diverse and structurally different from those of the CV sites. The rhizosphere-associated microbial communities were significantly affected by vegetation, soil physicochemical properties including EC, pH, and level of contaminants [35]. Spatial variability in rhizosphere microbial communities has been attributed mostly to different soil physicochemical characteristics, hydrodynamics and environmental contamination, as well as with localization of the vegetation [36]. For instance, when comparing two willow cultivars, the differences in the microbial communities between cultivars were relatively minor compared to the large differences between differentially contaminated soils [35]. The effect of environmental factors (such as soil moisture content, pH, EC, nutrients and contamination) is often stronger than the effect of vegetation type on the soil microbial communities [37].

Plant roots support larger populations of microorganism in their rhizosphere compared to non-rooted bulk soil [38, 39] by releasing organic material as root exudates [38], that increased microbial biomasses and their enzyme activities [40]. In the present work, the bacterial and fungal biomasses (PLFA abundance) were significantly higher in the vegetated sites compared to the un-vegetated sites; the superior levels of microbial biomass in the vegetated sites were also associated with greater rates of soil respiration and greater enzymatic activities. The increased urease and phosphatase activities in the rhizosphere-associated soil samples suggest that these soils may have increased mineralization rates of soil organic nitrogen and phosphorus compared to the unvegetated site. Similar to our findings, Yang et al [39] found significantly higher activities of β-glucosidase, urease and phosphatase in the rhizosphere soils of *Robinia pseudoacacia* compared to those of bulk soil. In addition, our results showed that the ratios of bacterial to fungal PLFAs increased in un-vegetated soil samples compared to those of vegetated soil samples. One possible reason for this could be that bacteria are better adapted to the site conditions than fungi, leading to more bacteria than fungi in the UV soil samples. Tardif et al. [35] also reported that the level of contamination had a more pronounced effect on willow plants’ rhizospheric fungal communities than on the bacterial communities.

The fungal diversity in the UV samples declined substantially compared to the CV, V-East, and V-West samples. A positive influence of plant presence on fungal diversity was significant in the V-East and V-West samples. The higher number of unidentified fungal OTUs seen in the CV, V-East, and V-West samples compared to the UV samples points to a closer association between plants and fungi. Similar to our study, a significant decline in fungal diversity was observed in highly contaminated soils where the introduction of willow plants significantly increased the fungal diversity [41]. Thion et al. [42] reported a positive effect of plants on fungal abundance and diversity in multi-contaminated soils over the long-term. These findings suggest that phytoremediation may have an unequal and direct impact on fungi in such contaminated environments, by stimulating fungi establishment. Compared to bacteria, less is known about fungal communities tolerance to petroleum-hydrocarbons [43].

Soil physiochemical analyses showed significant differences among the sampling locations. Samples from the CV and UV sites are part of SWMU/Shell Pond, which used to be filled with Bay water. This section was later drained, which resulted in the precipitation of salts leading to higher salinity. The two peripheral sites outside of Shell Pond have no history of water ponding and were colonized by a different pool of plant species. In the present study, localized soil physiochemical characteristic together with pickleweed plants colonization might have influenced the composition of bacterial and fungal communities. Oliveria [36] also reported that different physicochemical characteristics, hydrodynamics and environmental contamination, together with localization of the vegetation in the complex estuarine system of each sampling site, might have created partial variation in bacterial community composition. Electrical conductivity (a proxy for salinity) was the main physicochemical factor driving the changes in the composition of soil bacterial and fungal communities. Other studies also suggested that changes in the geoelectrical properties of the sediments create significant spatial heterogeneity in the structure of the microbial communities [44]. High salinity creates low osmotic potential that adversely affects the overall activities of microorganisms [45, 46], i.e., Qin et al. [46] reported that the drop in soil salinity from 2.86% to 0.10% resulted in 30% increase in the degradation rate of petroleum hydrocarbons. Apart from heavy metal contamination, soil EC along with pH and soil organic matter content were found to greatly influence the bacterial abundance and diversity associated with bulk soil [39].

Salinity and petroleum contamination exerted strong selective pressure to favor many potentially petroleum degrading and halophilic bacteria [47]. The bacterial group *Bacteroidetes*, responsible for bacterial diversity shift, was represented by the halophilic bacterial classes *Rhodothermi* (*Balneola*) and *Flavobacteriaceae* (*Salinimicrobium)*. Indeed, the presence of higher abundances of family *Balneolaceae* (*Bacteroidetes*) and halophilic Archaea represented by the genus *Haladaptatus* and *Halogranum* in the central CV and UV sites may be due to the selective pressure exerted by high salinity. Members belonging to the family *Balneolaceae* are halophilic bacteria mostly associated with alkaline areas [48, 49]. The class *Gammaproteobacteria* had similar abundances across the four sampling sites, both in the vegetated and un-vegetated sites. Other studies also reported the presence of *Gammaproteobacteria* in similar abundances in the low and high hydrocarbon contaminated sites [41]. Many members of *Cyanobacteria* are root associated symbionts involved mainly in biological nitrogen fixation and also produce plant growth promoting substances [50]. In our study, *Cyanobacteria* OTUs were only detected in the peripheral vegetated sites, which might be due to their sensitivity to high salinity in the central sites.

The implementation of phytoremediation and bioremediation strategies to petroleum-hydrocarbons-contaminated soils depends on biotic and abiotic factors of the site [51].The wide patterns of resource availability in the soil due to type and distribution patterns of plant species and soil physiochemical characteristics influence soil microbial community structure and diversity [52]. This study showed that the rhizospheric microbial communities differ in a contaminated soil compared to non-contaminated soil. Soil salinity in petroleum-contaminated sites is one of the major constraints to the biodegradation processes as it adversely affects soil physiochemical properties, crop productivity and microbiological activities to a significant extent [53–57].

## Supporting information

S1 Fig Relative abundance of alkanes, cycloalkanes, n-alkanes and PAHs at four sites within the SWMU. Error bars correspond to SEs (n = 8 for UV and CV, n = 4 for V-East and V-West).
(DOCX)

S2 Fig Relative abundance of carbon chain compounds of different C number extracted from the soils at peripheral vegetated V-East, V-West sites, central vegetated (CV) and central un-vegetated (UV) sites. Error bars correspond to SEs (n = 8 for UV and CV, n = 4 for V-East and V-West).
(DOCX)

S3 Fig Venn diagram showing the number of unique and shared bacterial (**A**) and fungal (**B**) operational taxonomic units at 97% genetic similarity associated with central SWMU sites; UV and CV and in the peripheral sites samples; V-East, and V-West.
(DOCX)

S1 Table F-values for pairwise PERMANOVA of OTU-based structure of bacterial communities at the peripheral vegetated sites; V-East and V-West, and in the central vegetated (CV) and un-vegetated (UV) sites. Values in bold denote statistical significance (p≤0.05), significance levels are shown at *p≤0.05, **p≤0.01 and ***p≤0.001.
(DOCX)

S2 Table R-values for pairwise ANOSIM of OTU-based structure of bacterial communities at the peripheral vegetated sites; V-East and V-West, and in the central vegetated (CV) and un-vegetated (UV) sites. Values in bold denote statistical significance (p≤0.05), significance levels are shown at *p≤0.05, **p≤0.01 and ***p≤0.001.
(DOCX)

S3 Table F-values for pairwise PERMANOVA of OTU-based structure of fungal communities in the peripheral vegetated sites; V-East and V-West, and in the central vegetated (CV) and un-vegetated (UV) sites. Values in bold denote statistical significance (p≤0.05), significance levels are shown at *p≤0.05, **p≤0.01 and ***p≤0.001.
(DOCX)

S4 Table R-values for pairwise ANOSIM of OTU-based structure of fungal communities at the peripheral vegetated sites; V-East and V-West, and in the central vegetated (CV) and un-vegetated (UV) sites. Values in bold denote statistical significance (p≤0.05), significance levels are shown at *p≤0.05, **p≤0.01 and ***p≤0.001.
(DOCX)

S5 Table Relative abundances (%) of the most abundant bacterial classes found at the peripheral vegetated sites; V-East and V-West, and at the central vegetated (CV) and un-vegetated (UV) sites. For each taxonomic group, mean values followed by different letters are significantly different (p ≤ 0.05) according to Tukey's HSD test.
(DOCX)

S6 Table Taxonomic classification of the top 50 OTUs contributing to dissimilarities (similarity percentage analysis) in bacterial community structures among the rhizosphere-associated soil from the investigated sites V-East, V-West and CV. OTUs were arrange in decreasing order of dissimilarity contribution.
(DOCX)

S7 Table Relative abundances (%) of the most abundant fungal classes found in the peripheral vegetated sites; V-East and V-West, and in the central vegetated (CV) and un-vegetated (UV) sites. For each taxonomic group, mean values followed by different letters are significantly different (p ≤ 0.05) according to Tukey's HSD test.
(DOCX)

S8 Table Taxonomic classification of the top 20 OTUs contributing to dissimilarities (similarity percentage analysis) in fungal community structures among the rhizosphere-associated soil from the investigated sites V-East, V-West and CV. OTUs were arrange in decreasing order of dissimilarity contribution.
(DOCX)
