# Supplementary material for: Variation in pickleweed root-associated microbial communities at different locations of a saline solid waste management unit contaminated with petroleum hydrocarbons
Source: PLoS One. 2019 Oct 3;14(10):e0222901. doi: 10.1371/journal.pone.0222901 (PMC6776359; doi:10.1371/journal.pone.0222901)
Supplement: S1 Fig — Error bars correspond to SEs (n = 8 for UV and CV, n = 4 for V-East and V-West). (DOCX) [file pone.0222901.s001.docx]

**
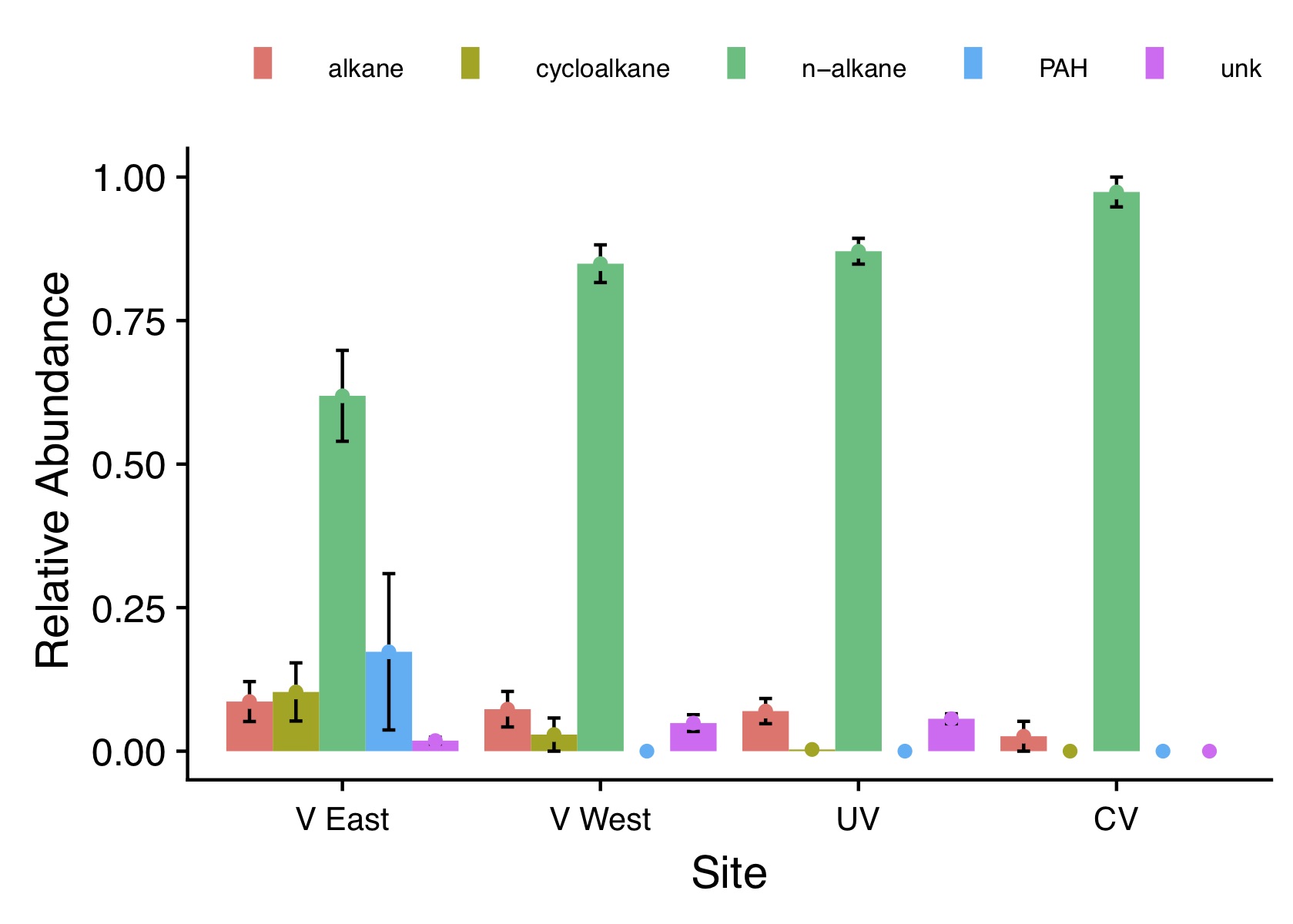
**

**S1 Fig.** Relative abundance of alkanes, cycloalkanes, n-alkanes and PAHs at four sites within the SWMU. Error bars correspond to SEs (n=8 for UV and CV, n=4 for V-East and V-West).
