# Supplementary material for: Variation in pickleweed root-associated microbial communities at different locations of a saline solid waste management unit contaminated with petroleum hydrocarbons
Source: PLoS One. 2019 Oct 3;14(10):e0222901. doi: 10.1371/journal.pone.0222901 (PMC6776359; doi:10.1371/journal.pone.0222901)
Supplement: S2 Fig — Error bars correspond to SEs (n = 8 for UV and CV, n = 4 for V-East and V-West). (DOCX) [file pone.0222901.s002.docx]

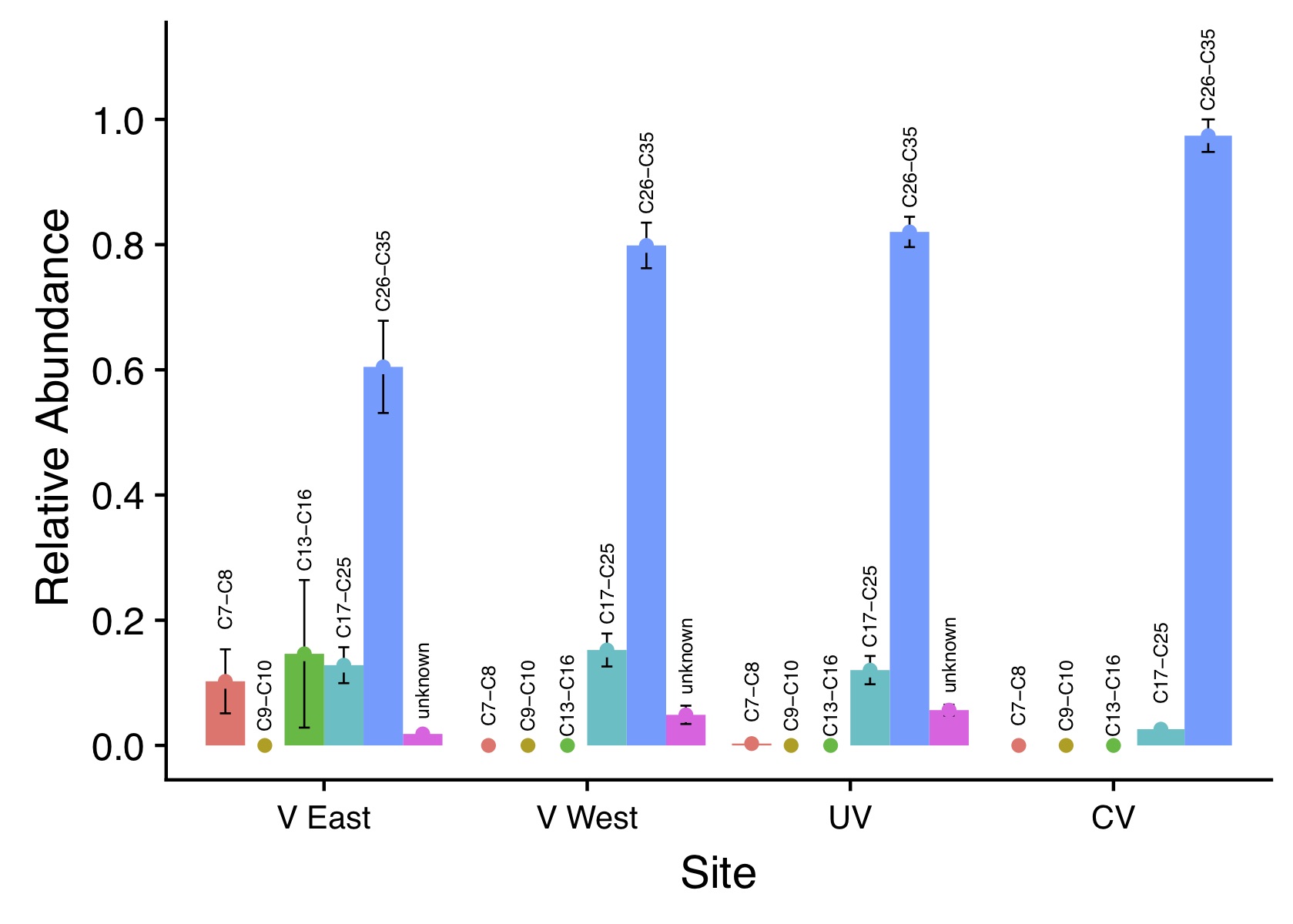


**S2 Fig.** Relative abundance of carbon chain compounds of different C number extracted from the soils at peripheral vegetated V-East, V-West sites, central vegetated (CV) and central un-vegetated (UV) sites. Error bars correspond to SEs (n=8 for UV and CV, n=4 for V-East and V-West).
