# Supplementary material for: Variation in pickleweed root-associated microbial communities at different locations of a saline solid waste management unit contaminated with petroleum hydrocarbons
Source: PLoS One. 2019 Oct 3;14(10):e0222901. doi: 10.1371/journal.pone.0222901 (PMC6776359; doi:10.1371/journal.pone.0222901)
Supplement: S3 Fig — Venn diagram showing the number of unique and shared bacterial (A) and fungal (B) operational taxonomic units at 97% genetic similarity associated with central SWMU sites; UV and CV and in the peripheral sites samples; V-East, and V-West. (DOCX) [file pone.0222901.s003.docx]

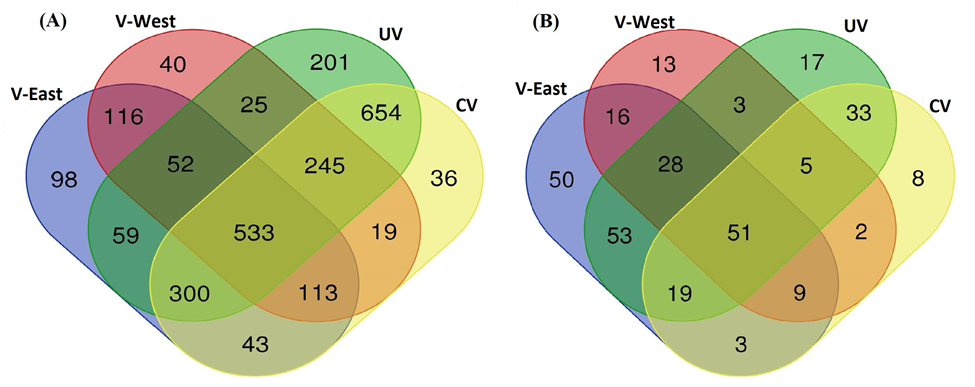


**S3 Fig.** Venn diagram showing the number of unique and shared bacterial **(A)** and fungal **(B)** operational taxonomic units at 97% genetic similarity associated with central SWMU sites; UV and CV and in the peripheral sites samples; V-East, and V-West.
