# Supplementary material for: Variation in pickleweed root-associated microbial communities at different locations of a saline solid waste management unit contaminated with petroleum hydrocarbons
Source: PLoS One. 2019 Oct 3;14(10):e0222901. doi: 10.1371/journal.pone.0222901 (PMC6776359; doi:10.1371/journal.pone.0222901)
Supplement: S3 Table — Values in bold denote statistical significance (p≤0.05), significance levels are shown at *p≤0.05, **p≤0.01 and ***p≤0.001. (DOCX) [file pone.0222901.s006.docx]

**S3 Table.** F-values for pairwise PERMANOVA of OTU-based structure of fungal communities in the peripheral vegetated sites; V-East and V-West, and in the central vegetated (CV) and un-vegetated (UV) sites. Values in bold denote statistical significance (p≤0.05), significance levels are shown at *p≤0.05, **p≤0.01 and ***p≤0.001.

|  | **V-East** | **V-West** | **CV** | **UV** |
| --- | --- | --- | --- | --- |
| **V-East** |  |  |  |  |
| **V-West** | **7.03*** |  |  |  |
| **CV** | **7.48**** | **9.58**** |  |  |
| **UV** | **9.18**** | **14.16**** | 1.62 |  |
