# Supplementary material for: Variation in pickleweed root-associated microbial communities at different locations of a saline solid waste management unit contaminated with petroleum hydrocarbons
Source: PLoS One. 2019 Oct 3;14(10):e0222901. doi: 10.1371/journal.pone.0222901 (PMC6776359; doi:10.1371/journal.pone.0222901)
Supplement: S4 Table — Values in bold denote statistical significance (p≤0.05), significance levels are shown at *p≤0.05, **p≤0.01 and ***p≤0.001. (DOCX) [file pone.0222901.s007.docx]

**S4 Table.** R-values for pairwise ANOSIM of OTU-based structure of fungal communities at the peripheral vegetated sites; V-East and V-West, and in the central vegetated (CV) and un-vegetated (UV) sites. Values in bold denote statistical significance (p≤0.05), significance levels are shown at *p≤0.05, **p≤0.01 and ***p≤0.001.

|  | **V-East** | **V-West** | **CV** | **UV** |
| --- | --- | --- | --- | --- |
| **V-East** |  |  |  |  |
| **V-West** | **0.895*** |  |  |  |
| **CV** | **0.998**** | **0.893**** |  |  |
| **UV** | **0.976**** | **0.853**** | 0.058 |  |
