# Supplementary material for: Variation in pickleweed root-associated microbial communities at different locations of a saline solid waste management unit contaminated with petroleum hydrocarbons
Source: PLoS One. 2019 Oct 3;14(10):e0222901. doi: 10.1371/journal.pone.0222901 (PMC6776359; doi:10.1371/journal.pone.0222901)
Supplement: S5 Table — For each taxonomic group, mean values followed by different letters are significantly different (p ≤ 0.05) according to Tukey's HSD test. (DOCX) [file pone.0222901.s008.docx]

**S5 Table.** Relative abundances (%) of the most abundant bacterial classes found at the peripheral vegetated sites; V-East and V-West, and at the central vegetated (CV) and un-vegetated (UV) sites. For each taxonomic group, mean values followed by different letters are significantly different (p ≤ 0.05) according to Tukey's HSD test.

| **Classes** | **V-East** | **V-West** | **CV** | **UV** |
| --- | --- | --- | --- | --- |
| *Rhodothermi* | 1.84 a | 5.67 ab | 20.05 c | 12.87 bc |
| *Flavobacteriia* | 3.02 a | 14.42 ab | 10.71 ab | 17.01 b |
| *Gammaproteobacteria* | 10.38 b | 3.51 a | 11.39 b | 12.75 b |
| *Alphaproteobacteria* | 9.63 a | 12.65 a | 8.45 a | 8.21 a |
| *Anaerolineae* | 4.00 a | 3.87 a | 5.97 a | 7.73 a |
| *Deltaproteobacteria* | 9.28 b | 4.58 a | 6.06 ab | 4.53 a |
| *Gemmatimonadetes* | 7.01 bc | 3.12 a | 4.40 ab | 7.53 c |
| *Cytophagia* | 5.82 ab | 8.76 b | 5.65 b | 2.53 a |
| *Bacteroidia* | 8.50 b | 0.50 a | 2.46 b | 4.68 b |
| *Betaproteobacteria* | 7.64 b | 2.60 a | 2.49 a | 2.62 a |
| *Planctomycetia* | 2.35 ab | 6.74 c | 2.49 b | 1.69 a |
| *Actinobacteria* | 3.48 a | 4.77 a | 1.71 a | 1.65 a |
| *Bacilli* | 0.27 a | 2.47 ab | 3.06 b | 1.72 ab |
| *Acidimicrobiia* | 2.88 b | 1.01 a | 1.35 ab | 1.74 ab |
| *Oscillatoriophycideae* | 0.03 b | 8.57 c | 0.00 a | 0.00 a |
| Others | 22.92 a | 14.33 a | 10.26 a | 12.44 a |
