# Supplementary material for: Variation in pickleweed root-associated microbial communities at different locations of a saline solid waste management unit contaminated with petroleum hydrocarbons
Source: PLoS One. 2019 Oct 3;14(10):e0222901. doi: 10.1371/journal.pone.0222901 (PMC6776359; doi:10.1371/journal.pone.0222901)
Supplement: S6 Table — OTUs were arrange in decreasing order of dissimilarity contribution. (DOCX) [file pone.0222901.s009.docx]

**S6 Table.** Taxonomic classification of the top 50 OTUs contributing to dissimilarities (similarity percentage analysis) in bacterial community structures among the rhizosphere-associated soil from the investigated sites V-East, V-West and CV. OTUs were arrange in decreasing order of dissimilarity contribution.

| OTU no. | Av. Diss. | Cont. % | Cum. % | Domain | Phylum | Class | Order | Family | Genus |
| --- | --- | --- | --- | --- | --- | --- | --- | --- | --- |
| OTU4 | 2.14 | 2.60 | 2.60 | Bacteria | *Cyanobacteria* | *Oscillatoriophycideae* | *Oscillatoriales* | *Phormidiaceae* | *Phormidium* |
| OTU3 | 0.98 | 1.20 | 3.80 | Bacteria | *Bacteroidetes* | *Rhodothermi* | *Rhodothermales* | *Balneolaceae* | KSA1 |
| OTU2 | 0.79 | 0.96 | 4.76 | Bacteria | *Bacteroidetes* | *Flavobacteriia* | *Flavobacteriales* | *Flavobacteriaceae* | Unclassified |
| OTU7 | 0.78 | 0.96 | 5.72 | Bacteria | *Bacteroidetes* | *Rhodothermi* | *Rhodothermales* | *Balneolaceae* | KSA1 |
| OTU1 | 0.70 | 0.85 | 6.57 | Bacteria | *Bacteroidetes* | *Flavobacteriia* | *Flavobacteriales* | *Flavobacteriaceae* | *Salinimicrobium* |
| OTU12 | 0.65 | 0.79 | 7.36 | Bacteria | *Bacteroidetes* | *Rhodothermi* | *Rhodothermales* | *Balneolaceae* | KSA1 |
| OTU23 | 0.64 | 0.78 | 8.14 | Bacteria | *Bacteroidetes* | *Bacteroidia* | *Bacteroidales* | Unclassified | Unclassified |
| OTU10 | 0.59 | 0.72 | 8.86 | Bacteria | *Bacteroidetes* | *Rhodothermi* | *Rhodothermales* | *Balneolaceae* | KSA1 |
| OTU6 | 0.54 | 0.66 | 9.53 | Bacteria | *Bacteroidetes* | *Rhodothermi* | *Rhodothermales* | *Balneolaceae* | *Balneola* |
| OTU35 | 0.43 | 0.53 | 10.05 | Bacteria | *Bacteroidetes* | *Flavobacteriia* | *Flavobacteriales* | *Flavobacteriaceae* | *Salinimicrobium* |
| OTU9 | 0.43 | 0.52 | 10.57 | Bacteria | *Bacteroidetes* | *Rhodothermi* | *Rhodothermales* | *Balneolaceae* | *Balneola* |
| OTU17 | 0.43 | 0.52 | 11.09 | Bacteria | *Bacteroidetes* | *Flavobacteriia* | *Flavobacteriales* | *Flavobacteriaceae* | *Salinimicrobium* |
| OTU73 | 0.40 | 0.49 | 11.58 | Bacteria | *Cyanobacteria* | *Synechococcophycideae* | *Pseudanabaenales* | *Pseudanabaenaceae* | Unclassified |
| OTU52 | 0.40 | 0.48 | 12.06 | Bacteria | *Proteobacteria* | *Gammaproteobacteria* | *Thiotrichales* | *Piscirickettsiaceae* | Unclassified |
| OTU24 | 0.38 | 0.47 | 12.53 | Bacteria | *Bacteroidetes* | *Flavobacteriia* | *Flavobacteriales* | *Flavobacteriaceae* | *Arenibacter* |
| OTU50 | 0.38 | 0.46 | 12.99 | Archaea | *Euryarchaeota* | *Halobacteria* | *Halobacteriales* | *Halobacteriaceae* | *Haladaptatus* |
| OTU72 | 0.37 | 0.45 | 13.43 | Bacteria | *Bacteroidetes* | *Cytophagia* | *Cytophagales* | *Flammeovirgaceae* | Unclassified |
| OTU85 | 0.36 | 0.44 | 13.87 | Bacteria | *Proteobacteria* | *Deltaproteobacteria* | *Myxococcales* | Unclassified | Unclassified |
| OTU20 | 0.36 | 0.44 | 14.31 | Bacteria | *Acidobacteria* | iii1-8 | DS-18 | Unclassified | Unclassified |
| OTU5 | 0.35 | 0.43 | 14.74 | Bacteria | *Bacteroidetes* | *Flavobacteriia* | *Flavobacteriales* | *Flavobacteriaceae* | *Salinimicrobium* |
| OTU89 | 0.35 | 0.43 | 15.16 | Bacteria | *Proteobacteria* | *Gammaproteobacteria* | *Thiotrichales* | *Piscirickettsiaceae* | Unclassified |
| OTU65 | 0.33 | 0.41 | 15.57 | Bacteria | *Actinobacteria* | *Acidimicrobiia* | *Acidimicrobiales* | Unclassified | Unclassified |
| OTU26 | 0.33 | 0.41 | 15.98 | Bacteria | *Bacteroidetes* | *Rhodothermi* | *Rhodothermales* | *Rhodothermaceae* | *Rubricoccus* |
| OTU92 | 0.32 | 0.39 | 16.36 | Bacteria | *Chlorobi* | *Ignavibacteria* | *Ignavibacteriales* | *Ignavibacteriaceae* | Unclassified |
| OTU53 | 0.32 | 0.39 | 16.75 | Bacteria | *Proteobacteria* | *Deltaproteobacteria* | *Desulfuromonadales* | *Pelobacteraceae* | Unclassified |
| OTU40 | 0.32 | 0.39 | 17.14 | Bacteria | *Bacteroidetes* | *Bacteroidia* | *Bacteroidales* | SB-1 | Unclassified |
| OTU46 | 0.32 | 0.39 | 17.53 | Bacteria | *Bacteroidetes* | *Rhodothermi* | *Rhodothermales* | *Balneolaceae* | KSA1 |
| OTU84 | 0.32 | 0.38 | 17.92 | Bacteria | *Bacteroidetes* | *Cytophagia* | *Cytophagales* | *Flammeovirgaceae* | Unclassified |
| OTU45 | 0.31 | 0.38 | 18.30 | Bacteria | *Cyanobacteria* | *Chloroplast* | *Stramenopiles* | Unclassified | Unclassified |
| OTU63 | 0.31 | 0.37 | 18.67 | Archaea | *Euryarchaeota* | *Halobacteria* | *Halobacteriales* | *Halobacteriaceae* | Haladaptatus |
| OTU33 | 0.30 | 0.37 | 19.04 | Bacteria | *Bacteroidetes* | *Rhodothermi* | *Rhodothermales* | *Balneolaceae* | Unclassified |
| OTU8 | 0.30 | 0.36 | 19.40 | Bacteria | *Bacteroidetes* | *Flavobacteriia* | *Flavobacteriales* | *Flavobacteriaceae* | *Salinimicrobium* |
| OTU18 | 0.30 | 0.36 | 19.76 | Bacteria | *Gemmatimonadetes* | *Gemmatimonadetes* | Unclassified | Unclassified | Unclassified |
| OTU55 | 0.29 | 0.36 | 20.12 | Bacteria | *Planctomycetes* | *Planctomycetia* | *Pirellulales* | *Pirellulaceae* | Unclassified |
| OTU29 | 0.29 | 0.35 | 20.47 | Bacteria | *Proteobacteria* | *Deltaproteobacteria* | *Myxococcales* | Unclassified | Unclassified |
| OTU19 | 0.28 | 0.35 | 20.81 | Bacteria | *Acidobacteria* | *Solibacteres* | *Solibacterales* | PAUC26f | Unclassified |
| OTU93 | 0.26 | 0.32 | 21.13 | Bacteria | *Bacteroidetes* | *Rhodothermi* | *Rhodothermales* | *Balneolaceae* | KSA1 |
| OTU110 | 0.26 | 0.32 | 21.45 | Bacteria | *Gemmatimonadetes* | *Gemmatimonadetes* | Unclassified | Unclassified | Unclassified |
| OTU96 | 0.26 | 0.32 | 21.76 | Bacteria | *Bacteroidetes* | *Cytophagia* | *Cytophagales* | *Flammeovirgaceae* | Unclassified |
| OTU34 | 0.26 | 0.31 | 22.08 | Bacteria | *Bacteroidetes* | *Rhodothermi* | *Rhodothermales* | *Balneolaceae* | KSA1 |
| OTU11 | 0.25 | 0.31 | 22.38 | Bacteria | *Bacteroidetes* | *Rhodothermi* | *Rhodothermales* | *Balneolaceae* | *Balneola* |
| OTU107 | 0.24 | 0.29 | 22.67 | Bacteria | *Bacteroidetes* | *Cytophagia* | *Cytophagales* | *Flammeovirgaceae* | Unclassified |
| OTU37 | 0.23 | 0.28 | 22.96 | Bacteria | *Bacteroidetes* | *Bacteroidia* | *Bacteroidales* | Unclassified | Unclassified |
| OTU64 | 0.23 | 0.28 | 23.24 | Bacteria | *Chloroflexi* | *Anaerolineae* | *Caldilineales* | *Caldilineaceae* | Unclassified |
| OTU21 | 0.23 | 0.28 | 23.52 | Bacteria | *Chloroflexi* | *Anaerolineae* | CFB-26 | Unclassified | Unclassified |
| OTU13 | 0.23 | 0.28 | 23.80 | Bacteria | *Bacteroidetes* | *Bacteroidia* | *Bacteroidales* | SB-1 | Unclassified |
| OTU49 | 0.23 | 0.28 | 24.07 | Archaea | *Euryarchaeota* | *Halobacteria* | *Halobacteriales* | *Halobacteriaceae* | *Halogranum* |
| OTU88 | 0.22 | 0.27 | 24.34 | Bacteria | *Gemmatimonadetes* | *Gemmatimonadetes* | Unclassified | Unclassified | Unclassified |
| OTU15 | 0.22 | 0.27 | 24.62 | Bacteria | *Proteobacteria* | *Alphaproteobacteria* | *Rhizobiales* | Unclassified | Unclassified |
| OTU16 | 0.22 | 0.27 | 24.89 | Bacteria | *Gemmatimonadetes* | *Gemmatimonadetes* | Unclassified | Unclassified | Unclassified |
