# Supplementary material for: Variation in pickleweed root-associated microbial communities at different locations of a saline solid waste management unit contaminated with petroleum hydrocarbons
Source: PLoS One. 2019 Oct 3;14(10):e0222901. doi: 10.1371/journal.pone.0222901 (PMC6776359; doi:10.1371/journal.pone.0222901)
Supplement: S7 Table — For each taxonomic group, mean values followed by different letters are significantly different (p ≤ 0.05) according to Tukey's HSD test. (DOCX) [file pone.0222901.s010.docx]

**S7 Table.** Relative abundances (%) of the most abundant fungal classes found in the peripheral vegetated sites; V-East and V-West, and in the central vegetated (CV) and un-vegetated (UV) sites. For each taxonomic group, mean values followed by different letters are significantly different (p ≤ 0.05) according to Tukey's HSD test.

| **Classes** | **V-East** | **V-West** | **CV** | **UV** |
| --- | --- | --- | --- | --- |
| *Sordariomycetes* | 20.37 a | 19.62 a | 54.43 b | 51.41 b |
| *Dothideomycetes* | 36.49 b | 57.70 b | 8.39 a | 7.78 a |
| *Agaricomycetes* | 0.21 a | 0.18 a | 25.25 b | 33.89 b |
| *Leotiomycetes* | 12.15 b | 0.35 a | 0.08 a | 0.53 a |
| *Chytridiomycetes* | 0.23 a | 7.66 a | 0.07 a | 0.06 a |
| *Eurotiomycetes* | 0.24 a | 0.15 a | 0.19 a | 1.06 a |
| Others | 3.93 a | 0.83 a | 0.02 a | 0.50 a |
| Unidentified | 26.38 b | 13.52 b | 11.57 b | 4.76 a |
