# Supplementary material for: Variation in pickleweed root-associated microbial communities at different locations of a saline solid waste management unit contaminated with petroleum hydrocarbons
Source: PLoS One. 2019 Oct 3;14(10):e0222901. doi: 10.1371/journal.pone.0222901 (PMC6776359; doi:10.1371/journal.pone.0222901)
Supplement: S8 Table — OTUs were arrange in decreasing order of dissimilarity contribution. (DOCX) [file pone.0222901.s011.docx]

**S8 Table.** Taxonomic classification of the top 20 OTUs contributing to dissimilarities (similarity percentage analysis) in fungal community structures among the rhizosphere-associated soil from the investigated sites V-East, V-West and CV. OTUs were arrange in decreasing order of dissimilarity contribution.

| OTU no. | Av. dissim | Contrib. % | Cumulative % | Phlym | Class | Order | Family | Genus |
| --- | --- | --- | --- | --- | --- | --- | --- | --- |
| OTU1 | 11,47 | 13,01 | 13,01 | *Ascomycota* | *Sordariomycetes* | *Microascales* | *Microascaceae* | *Scedosporium* |
| OTU2 | 8,949 | 10,15 | 23,16 | *Ascomycota* | *Sordariomycetes* | *Sordariales* | *Chaetomiaceae* | Unidentified |
| OTU3 | 7,783 | 8,829 | 31,99 | *Ascomycota* | *Dothideomycetes* | *Pleosporales* | *Pleosporaceae* | *Alternaria* |
| OTU4 | 5,2 | 5,899 | 37,89 | *Ascomycota* | *Dothideomycetes* | *Capnodiales* | *Davidiellaceae* | *Acroconidiella* |
| OTU11 | 4,575 | 5,189 | 43,08 | *Ascomycota* | *Dothideomycetes* | *Pleosporales* | Incertae_sedis | *Phoma* |
| OTU7 | 4,519 | 5,127 | 48,2 | *Ascomycota* | *Dothideomycetes* | *Pleosporales* | *Pleosporaceae* | *Macrospora* |
| OTU12 | 3,412 | 3,87 | 52,07 | *Ascomycota* | *Leotiomycetes* | *Helotiales* | Incertae_sedis | *Scytalidium* |
| OTU10 | 2,686 | 3,047 | 55,12 | Unidentified | Unidentified | Unidentified | Unidentified | Unidentified |
| OTU8 | 2,544 | 2,886 | 58,01 | *Basidiomycota* | *Agaricomycetes* | Incertae_sedis | Incertae_sedis | *Myriococcum* |
| OTU5 | 2,381 | 2,701 | 60,71 | *Basidiomycota* | *Agaricomycetes* | *Agaricales* | Unidentified | Unidentified |
| OTU17 | 2,305 | 2,614 | 63,32 | *Chytridiomycota* | *Chytridiomycetes* | *Rhizophydiales* | *Rhizophydiaceae* | *Rhizophydium* |
| OTU6 | 2,104 | 2,386 | 65,71 | *Basidiomycota* | *Agaricomycetes* | *Agaricales* | *Coprinaceae* | *Coprinus* |
| OTU15 | 2,027 | 2,299 | 68,01 | Unidentified | Unidentified | Unidentified | Unidentified | Unidentified |
| OTU18 | 1,995 | 2,263 | 70,27 | *Ascomycota* | *Sordariomycetes* | *Hypocreales* | Unidentified | Unidentified |
| OTU9 | 1,601 | 1,816 | 72,09 | *Basidiomycota* | *Agaricomycetes* | *Agaricales* | *Psathyrellaceae* | *Psathyrella* |
| OTU16 | 1,501 | 1,703 | 73,79 | *Ascomycota* | *Sordariomycetes* | *Hypocreales* | *Nectriaceae* | *Fusarium* |
| OTU14 | 1,246 | 1,414 | 75,2 | Unidentified | Unidentified | Unidentified | Unidentified | Unidentified |
| OTU19 | 1,167 | 1,323 | 76,52 | *Ascomycota* | *Sordariomycetes* | *Phyllachorales* | *Phyllachoraceae* | Unidentified |
| OTU20 | 1,063 | 1,206 | 77,73 | *Ascomycota* | *Dothideomycetes* | *Pleosporales* | *Pleosporaceae* | *Alternaria* |
| OTU13 | 0,956 | 1,084 | 78,82 | *Basidiomycota* | *Agaricomycetes* | *Agaricales* | Unidentified | Unidentified |
